# Continuous versus Conventional Infusion of Amphotericin B Deoxycholate: A Meta-Analysis

**DOI:** 10.1371/journal.pone.0077075

**Published:** 2013-10-21

**Authors:** Matthew E. Falagas, Drosos E. Karageorgopoulos, Giannoula S. Tansarli

**Affiliations:** 1 Alfa Institute of Biomedical Sciences (AIBS), Athens, Greece; 2 Department of Internal Medicine - Infectious Diseases, Mitera Hospital, Hygeia Group, Athens, Greece; 3 Department of Medicine, Tufts University School of Medicine, Boston, Massachusetts, United States of America; Cardiff University, United Kingdom

## Abstract

**Background:**

Treatment with Amphotericin B (AmB) deoxycholate, which is still used widely, particularly in low-resource countries, has been challenged due to nephrotoxicity. We sought to study whether continuous infusion of AmB deoxycholate reduces nephrotoxicity retaining, however, the effectiveness of the drug.

**Methods:**

PubMed and Scopus databases were systematically searched to identify studies comparing the outcomes of patients receiving 24-h infusion of AmB (“continuous group”) and those receiving 2–6-h infusion of AmB (“conventional group”). Nephrotoxicity and all-cause mortality were the primary outcomes of the review, while treatment failure was the secondary outcome.

**Results:**

Five studies met the inclusion criteria; one randomized controlled trial, two prospective cohort studies, and two retrospective cohort studies. The majority of patients were neutropenic with an underlying hematologic malignancy. All 5 studies (392 patients) provided data regarding the development of nephrotoxicity. A non-significant trend towards lower nephrotoxicity was observed for patients receiving continuous infusion of AmB compared with those receiving conventional infusion [RR = 0.61 (95% CI 0.36, 1.02)]. Four studies (365 patients) provided data regarding mortality; no relevant difference was detected between patients receiving continuous and those receiving conventional infusion of AmB [RR = 0.81 (95% CI 0.36, 1.83)]. Data on treatment failure of the two methods of administration was insufficient for meaningful conclusions.

**Conclusion:**

The available evidence from mainly non-randomized studies suggests that continuous infusion of AmB deoxycholate might offer an advantage over the conventional infusion regarding the development of nephrotoxicity, without compromising patient survival. Further randomized studies are needed to investigate this issue.

## Introduction

The incidence of fungal infections has been increasing during the last decades. Amphotericin B (AmB) deoxycholate (“conventional” or crystalline AmB), a polyene, is one of the “oldest” (introduced in 1959), but still an established antifungal agent for the treatment of various invasive fungal infections (IFIs). It is for example the cornerstone for the treatment of crytpococcosis among patients with human immunodeficiency virus (HIV) infection and is widely used in regions with high prevalence of HIV infection, like Africa. [1], [2] Furthermore, AmB along with fluconazole and flucytosine have an important role in lower urinary tract infections due to achievement of high concentrations in the urine, in contrast to other antifungals. [3] In addition, apart from the parenteral administration, AmB can also be used locally for bladder irrigation [3].

However, the utility of AmB deoxycholate has been challenged due to the frequent occurrence of adverse events, including mainly infusion-related adverse events and nephrotoxicity. [4] Lipid-based formulations of AmB have been developed to provide a better safety profile. With the main exception of liposomal AmB for disseminated histoplasmosis in AIDS patients, the lipid-based formulations of AmB have not conclusively shown superior clinical effectiveness in terms of mortality compared with AmB deoxycholate. [5] The reduction in adverse events, particularly nephrotoxicity, which can be gained with the use of lipid-based formulations of AmB comes at significantly increased costs.

New broad-spectrum antifungals, like echinocandins and new generation azoles, have been introduced into clinical practice during the last 15 years for the treatment of severe infections caused by pathogens such as *Candida* spp. *Aspergillus* spp. [6], [7], [8] A meta-analysis of randomized controlled trials (RCTs) has suggested that patients who were treated with an echinocandin namely caspofungin had significantly higher clinical cure and fewer adverse events than those treated with AmB for infections caused by *Candida* spp.; however, no difference in mortality was detected between the compared groups [9].

IFIs are characterized by high mortality, [10], [11], [12] while most occur in patients with hematologic malignancies, transplant recipients, critically ill patients, or patients with HIV infection. [13] The introduction of new antifungal agents does not seem to decrease mortality, while their high cost prohibits the use in countries with low financial resources. The use of AmB deoxycholate is limited by the induced nephrotoxicity. Therefore, new methods of administration of the existing drugs could be considered.

A recently published meta-analysis showed that patients with severe bacterial infections who received extended or continuous infusion of carbapenems or piperacillin/tazobactam had significantly lower mortality than patients who received conventional short-term infusion of the antibiotics. [14] Although AmB is an “old” drug, its pharmacokinetic and pharmacodynamic properties have not been adequately clarified. It has been shown that concentration-dependent properties may overmatch the time-dependent ones. [15], [16], [17] Evidence from *in vitro* and *in vivo* data regarding the impact of time-dependent pharmacodynamics of AmB on outcomes is scarce.

In this context, we sought to systematically review and synthesize the available evidence, with the method of the meta-analysis, in order to examine whether 24-h (continuous) infusion of AmB deoxycholate can result in improved safety compared with infusion of conventional duration, without a compromise in clinical effectiveness.

## Methods

### Literature Search

We performed a systematic search in PubMed and Scopus databases through January 2013. The following search term was applied to all published articles in the PubMed database: “(antifungal OR amphotericin) AND (extended OR prolonged OR continuous OR intermittent OR short OR bolus) AND (infusion) AND (cure OR failure OR success OR effectiveness OR efficacy OR mortality OR died OR improve or randomized or trial)”. The following search term was applied to all published articles in the Scopus database: “(antifungal OR amphotericin) AND (extended OR prolonged OR continuous OR intermittent OR short OR bolus) AND (infusion) AND (cure OR failure OR success OR effectiveness OR efficacy OR mortality OR died OR improve)”. Hand-searching was also performed in the bibliographies of relevant articles so that additional potentially eligible studies could be retrieved. Articles published in languages other than English, French, German, Spanish, Italian, or Greek were not evaluated.

### Study Selection

Both randomized clinical trials and non-randomized comparative studies evaluating the clinical outcome between patients with fungal infections receiving continuous infusion and patients receiving conventional infusion of AmB were considered eligible for inclusion in this systematic review. Single-arm studies reporting the clinical outcomes of patients receiving either continuous or conventional infusion of AmB were excluded. Studies comparing the clinical outcomes between patients receiving dosing regimens of AmB other than the aforementioned were also excluded.

### Data Extraction

The extracted data consisted of the main characteristics of each study (first author, publication year, study design, period, and country), patient population, number of patients treated with AmB, and dosing regimen of AmB.

### Definitions and Outcomes

Two patient groups were considered for the purpose of the review: the “continuous infusion” group which comprised patients who received 24-h infusion of AmB, and the “conventional infusion” group comprising patients who received infusions of AmB lasting from 2 to 6 hours. Nephrotoxicity was defined according to the criteria used by the investigators of each study. The number of patients with electrolyte abnormalities, if provided separately from nephrotoxicity, was not added to the total number of patients with nephrotoxicity, but was presented separately to avoid potential overlapping. Acute adverse events occurring during administration of AmB were defined as infusion-related and included chills or rigors, fever, nausea, vomiting, exanthema, headache, and phlebitis.

The primary outcomes of this review were nephrotoxicity and all-cause mortality, while secondary outcome was treatment failure, among patients with fungal infections receiving continuous and those receiving conventional infusion of AmB. Treatment failure was defined according to the definitions used by the investigators of the original studies.

### Statistical Analysis

Pooled risk ratios (RR) and 95% confidence intervals (CI) were calculated using a Mantel-Haenszel fixed effect model (FEM) when there was no significant statistical heterogeneity between the studies; otherwise, the random effects model (REM) was used. Statistical heterogeneity between studies was assessed by the *χ* ^2^ test (p<0.10 indicates the presence of heterogeneity) and the *I* ^2^ (for assessing the degree of heterogeneity). The meta-analysis was performed with Review Manager for Windows, version 5.1 (The Nordic Cochrane Center of the Cochrane Collaboration, Copenhagen, Denmark).

## Results

**Figure 1:**
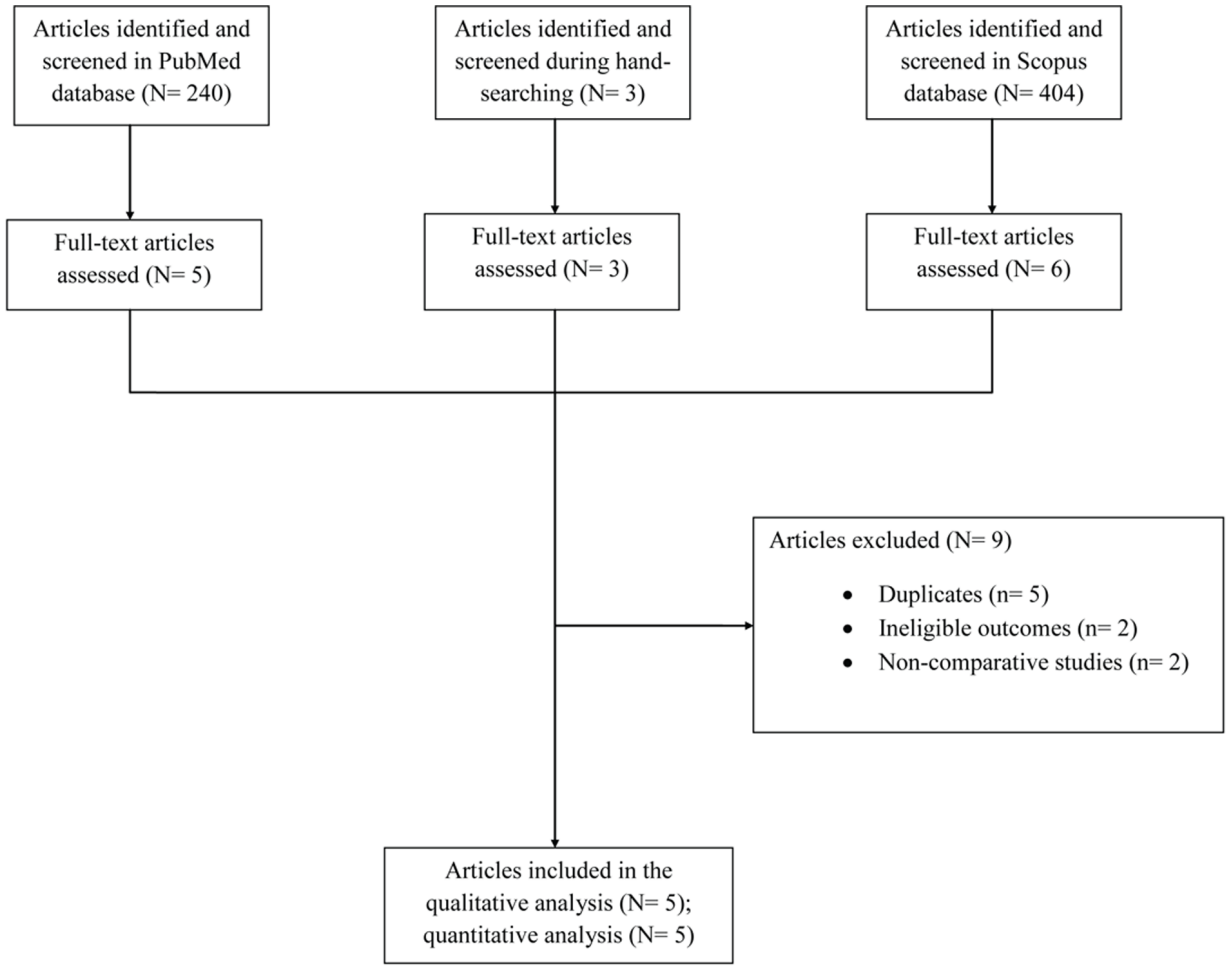
Flow diagram of the detailed study selection process.

A total of 647 articles were retrieved during the search process in both databases (240 articles from PubMed, 404 articles from Scopus, 3 from hand-searching). Five studies, that evaluated a total of 392 patients, were included in the review. [18], [19], [20], [21], [22] The study was written according to the PRISMA statement for systematic reviews and meta-analyses. The detailed search process and study selection is depicted in Figure 1. One study was RCT, [19] two were prospective cohort studies, [20], [22] and the remaining two were retrospective cohort studies. [18], [21] In 4 studies, all patients were neutropenic, [18], [19], [21], [22] while in the remaining study, 63% of the patients were neutropenic. [20] The vast majority of the included patients in all studies had a hematologic malignancy. Four out of the five studies reported the causative fungi that were identified. [19], [20], [21], [22] *Aspergillus* spp. was reported in all 4 studies, [19], [20], [21], [22] *Candida* spp. in 3 studies, [19], [20], [21] and *Cryptococcus* spp. in 2 studies. [19], [20] Ninety-three out of the 392 included patients (23.7%) had a proven IFI. [19], [20], [21] Four out of five of the included studies did not provide the daily dosage of AmB that was administered to the compared groups; [18], [19], [20], [21] therefore, a comparison of the amount of the administered drug between the two groups was unfeasible. In the one study that provided relevant data, the daily dosage of AmB was not the same between the compared groups. [22] Table 1 outlines the main characteristics of the included studies.

**Table 1:** Main characteristics of the included studies.

First author; Year	Study design; period, country	Patient population; number of pts treated with AmB (continuous versus conventional)	Reported fungal infections	Dosing regimen of AmB(continuous versusconventional)	Patients with renal and infusion-related adverse events, % (continuous versus conventional)	Definitions used for nephrotoxicity
Altmannsberger; 2007^18^	Retrospective cohort; NR, Germany	Neutropenic pts at a high risk of fungal infection: subjected to allogenic stem cell transplantation or to aggressive cytostatic treatment leading to neutropenia of >10 d; 56 (36 vs 20)	NR	24-h vs 6-h	Renal impairment	According to CTC
					CTC grade I: 50% vs 60%	
					CTC grade II: 22% vs 0%	
					CTC grade III: 0% vs 5%	
					Chills and exanthema: 14% vs 10%	
Eriksson; 2001^19^	RCT; NR, Switzerland	Neutropenic pts due to hematologic malignancies (90%) or other diseases (10%); 80 (40 vs 40)	Candidiasis, aspergillosis, cryptococcosis, *Rhizopus pusillus* infection	24-h vs 4-h	Neprotoxicity*: 48% vs 95%	Creatinine concentration >1.5 times baseline value
					Hypokalaemia: 10% vs 25%	
					Hypomagnesaemia: 43% vs 48%	
					Hypernatraemia: 5% vs 8%	
					Chills or rigors: 20% vs 63%	
					Vomiting: 28% vs 60%	
					Headache: 10% vs 28%	
					Others: 5% vs 20%	
Maharom; 2006^20^	Prospective cohort; .2004–2006, Thailand	Pts who needed AmB (66% with hematologic malignancy, 63% with neutropenia with refractory fever); 148 (91 vs 57)	Cryptococcosis, aspergillosis, *Penicillium* infection, histoplasmosis, mucormycosis, candidaemia, *Cladophialophora bantiana* infection	24-h vs 4–6-h	Nephrotoxicity: 28% vs 39%	Renal impairment was defined as a doubling of baseline serum creatinine
					Chills: 7% vs 27%	
					Nausea: 7% vs 13%	
					Phlebitis: 23% vs 19%	
Peleg; 2004^21^	Retrospectivecohort;2001–2003,Australia	Pts with refractory fever during neutropenia or possible/probable/proven fungal infection due to hematologic malignancy (99%); 81 (39 vs 42)	Candidaemia, aspergillosis, *Trichosporon beigelii* infection, *Exserohilum rostratum* infection	24-h vs 4-h	Renal impairment: 10% vs 45%	Renal impairment was defined as a doubling of baseline serum creatinine (creatinine ratio >2)
Schulenburg; 2005^22^	Prospective cohort with historical control; 1998–2003, Austria	Neutropenic pts due to hematologic malignancies with infections;27 (17 vs 10)	Aspergillosis	24-h vs 2–6-h	Nephrotoxicity: 0% vs 0%	According to WHO criteria
					Hypokalaemia: 88% vs 80%	
					Chills: 0% vs 20%	
					Drug fever: 0% vs 20%	

* In this study, nephrotoxicity was not defined by the investigators. For the purpose of our study, we considered as nephrotoxicity the number of patients who had ≥1.5 times baseline creatinine serum concentration. The number of patients with hypokalaemia was not added, because many of them may have been also included in those with elevated serum concentration creatinine.

**Abbreviations** AmB: amphotericin B, RCT: randomized controlled trial, pts: patients, CTC: common toxicity criteria, WHO: world health organization.

### Nephrotoxicity

Five studies provided data regarding nephrotoxicity [18], [19], [20], [21], [22] and in two of them, the compared treatment groups had a significant difference in nephrotoxicity. More specifically, patients receiving continuous infusion of AmB had significantly lower nephrotoxicity compared to patients receiving conventional infusion of AmB, [RR = 0.50, (95% CI: 0.36, 0.70)] and [RR = 0.23, (95% CI: 0.08, 0.61)] in the two studies respectively [19], [21].

**Figure 2:**
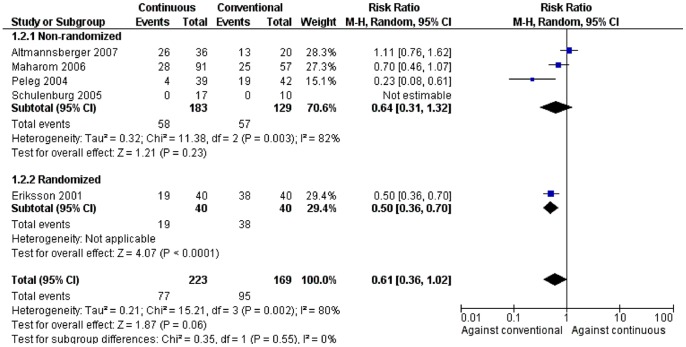
Forest plot depicting the risk ratios (RR) of nephrotoxicity for patients receiving continuous versus conventional infusion of amphotericin B deoxycholate, stratified by non-randomized and randomized studies. (Vertical line = “no difference” point between the two regimens. Squares = risk ratios; Diamonds = pooled risk ratios for all studies. Horizontal lines = 95% confidence intervals).

In the pooled analysis of the 5 studies no significant difference in nephrotoxicity could be shown between patients receiving continuous infusion of AmB and those receiving conventional infusion, but the lack of statistical significance was borderline [Figure 2, 392 patients, RR = 0.61 (95% CI: 0.36, 1.02)]. Significantly lower nephrotoxicity was observed in the continuous infusion group than the conventional infusion group in the subgroup of randomized studies which included only one study [Figure 2, 80 patients, RR = 0.50, (95% CI: 0.36, 0.70)]. Overall, high between-studies statistical heterogeneity was detected in this analysis (*I* ^2^ = 80%).

All but one of the included studies provided data with regard to infusion-related adverse events which mainly consisted of chills and gastrointestinal disorders. [18], [19], [20], [22] However, pooling of these data was unfeasible because different types of adverse events were reported in each study. Chills were significantly less common among patients receiving continuous infusion of AmB compared to those receiving conventional infusion in three studies. [19], [20], [22] In addition, vomiting and drug fever were also significantly less common among patients receiving continuous infusion of AmB than those receiving conventional infusion of AmB in two studies, respectively. [19], [22] Last, one study reported that infusion-related adverse events, including chills, were more common among patients who were treated with continuous infusion of AmB than those who were treated with conventional infusion, without, however, presenting a statistical analysis [18].

### Mortality

Four studies reported data on all-cause mortality [18], [19], [20], [21] and in two of them, a statistically significant difference was found between the compared groups. [20], [21] Both studies were non-randomized comparative studies. Although all-cause mortality significantly differed between the two compared treatment groups in both of them, there was no difference in mortality related to fungal infections in any of them. Specifically, the one study showed that patients receiving continuous infusion of AmB had lower mortality than those receiving conventional infusion. [21] On the contrary, in the other study, patients who were treated with continuous infusion of AmB had higher mortality compared to those who were treated with conventional infusion. [20] Moreover, in this latter study, the multivariate analysis did not show association between continuous infusion of AmB and mortality (p = 0.099).

**Figure 3:**
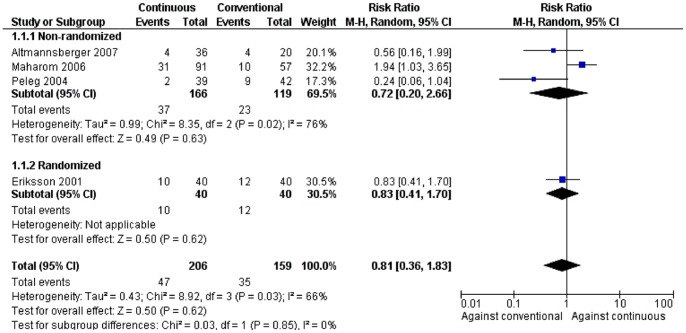
Forest plot depicting the risk ratios (RR) of mortality for patients receiving continuous versus conventional infusion of amphotericin B deoxycholate, stratified by non-randomized and randomized studies. (Vertical line = “no difference” point between the two regimens. Squares = risk ratios; Diamonds = pooled risk ratios for all studies. Horizontal lines = 95% confidence intervals).

In the pooled analysis of the 4 studies, no difference was found regarding mortality between patients receiving continuous and those receiving conventional infusion of AmB [Figure 3, 365 patients, RR = 0.81 (95% CI: 0.36, 1.83)]. The finding was verified both in the analysis of non-randomized studies [Figure 3, 285 patients, RR = 0.72 (95% CI: 0.20, 2.66)] and in the only randomized study [Figure 3, 365 patients, RR = 0.83 (95% CI: 0.41, 1.70)]. Overall, substantial statistical heterogeneity was observed in this analysis (*I* ^2^ = 66%).

### Treatment Failure

Two studies (229 patients) provided data on treatment failure. [20], [21] In one of these studies, 2.9% of the patients who were treated with continuous infusion of AmB had treatment failure, while no patients treated with conventional infusion of AmB experienced treatment failure. [20] In the other study, treatment failure was observed in 23% of the patients who were treated with continuous infusion of AmB, while the corresponding figure was 30% for the patients treated with conventional infusion of AmB [21].

## Discussion

The main finding of this meta-analysis is that the patients receiving AmB deoxycholate in a continuous infusion had a trend for lower nephrotoxicity than the patients receiving a conventional infusion. The continuous infusion of AmB for IFIs in hematological and mostly neutropenic patients did not appear to be associated with an increase in mortality compared with the conventional infusion. The high statistical heterogeneity observed in the analyses for nephrotoxicity and mortality is possibly due to the non-randomized design of the 4 out of 5 the included studies. Data regarding treatment failure in the included studies was not sufficient to draw a safe conclusion.

It is noteworthy that the only randomized study included showed significantly lower nephrotoxicity with the continuous infusion of AmB deoxycholate compared to conventional infusion. Although the findings of this trial are more credible compared with those of the non-randomized comparative studies, the latter have failed to find such a difference. The inclusion of non-randomized data means that the effect of potentially confounding factors, such the severity of the patients, could not be adjusted for. Also, there is room for bias in the interpretation of the outcomes of the analyses. However, this is the available evidence so far on this issue, and conclusively randomized studies need to further address this clinical question.

AmB deoxycholate is approved to be administered in an extended infusion lasting 2–6 hours because, infusion-related adverse events decrease through this method of administration compared with bolus infusion. [23], [24] A meta-analysis on the adverse events of the antifungal agents used against IFIs has shown that AmB deoxycholate is the most nephrotoxic AmB formulation, while the lipid formulations are less nephrotoxic. [25] Several lines of evidence from pharmacokinetic and non-comparative clinical studies have suggested that continuous infusion of AmB deoxycholate might reduce the nephrotoxicity of the drug [26], [27], [28].

Our review also showed that the infusion-related adverse events reported in the included studies seemed to be less common with the continuous than the conventional infusion of AmB. However, no uniform definitions for these adverse events were used in the included studies and the available data were not appropriate for a meta-analysis. Additionally, the meta-analysis showed that continuous infusion of AmB might be safer regarding the development of nephrotoxicity than the conventional infusion of AmB. Accordingly, the considerations regarding the safety of the drug could be partially overcome by using a continuous infusion of AmB.

Fungal infections have a high incidence among patients with prolonged neutropenia due to malignancies, mainly hematologic ones. Mortality in these populations is high and fungal infections are ranked among the top causes of death. Apart from safety, clinical effectiveness is an equally important reason for which a novel method of administration of a drug should be adopted in clinical practice. The present meta-analysis did not show that the conventional infusion of AmB in neutropenic patients has a comparative advantage over continuous infusion in terms of mortality, while data on treatment failure was insufficient.

The pharmacokinetic and pharmacodynamic properties of AmB deoxycholate could provide further insight into our observations. It appears that, *in vitro,* higher concentrations of the drug result in better fungicidal activity, while lower concentrations can be fungistatic. This is because higher drug concentrations can lead to greater disruption of the fungal cell membrane through binding to ergosterol. Higher AmB concentrations can, however, result in greater toxicity for humans by binding to sterols in the mammalian cell membrane.

The conventional 2–4 h infusion of AmB deoxycholate results in higher peak plasma total drug concentrations compared with the continuous infusion. However, the difference in the free drug concentrations between the two modes of administration may not be pronounced. [29] Higher total plasma AmB concentrations result in a greater fraction of protein-binding, due to the poor solubility of the drug. [30] High protein-binding appears to limit the bioactivity of the drug in tissues, resulting in fungistatic rather than fungicidal levels. [31] For these reasons, administering the drug in a continuous infusion might not result in an important reduction in the free-drug concentration in tissues compared with the conventional 2–4 h infusion. Higher peak total drug concentrations can, on the other hand, result in greater distribution of the drug in kidneys, potentially leading to higher nephrotoxicity. [29] The better toxicity profile associated with the continuous infusion of AmB deoxycholate can allow for the total daily dose of the drug to be increased in the case of serious infections [32].

Certain limitations should be considered for the interpretation of the findings of our meta-analysis. First, the non-randomized design of the included studies warrants caution in the interpretation of the findings of this meta-analysis due to the methodological issues reported above, while different definitions for nephrotoxicity were used among the included studies. However, it should be noted that there was satisfactory clinical homogeneity in the characteristics of the included patients, since the vast majority of them were neutropenic with an underlying hematologic malignancy. In addition, the total number of the included patients was rather small. Furthermore, we could not conclude whether there is difference in the effectiveness of the treatment with continuous versus conventional infusion of AmB in studies where a higher number of IFIs were documented compared with those that had a lower number of infections. A small percentage of the included patients had a proven IFI because the firm diagnosis of these infections is generally difficult in routine clinical practice and most of the patients are treated empirically or preemptively. Last, fungal infections differed among the included studies regarding the causative pathogens.

In conclusion, the continuous infusion of AmB deoxycholate might be a strategy for improving the safety of AmB administration, since our meta-analysis, which included mainly non-randomized studies, showed a non-significant trend towards lower nephrotoxicity with the continuous infusion of AmB deoxycholate than with the conventional infusion. The continuous infusion of AmB does not appear to entail a compromise in terms of mortality for hematological malignancy patients with IFIs. Future randomized studies should aim to corroborate the above observations, given the importance of amphotericin B for our armamentarium against IFIs, and that the use of lipid-based formulations is limited by the associated high costs in many healthcare settings.

## Supporting Information

Checklist S1 **PRISMA Checklist.**
(DOC)
